# Gα12 and endoplasmic reticulum stress-mediated pyroptosis in a single cycle of dextran sulfate-induced mouse colitis

**DOI:** 10.1038/s41598-024-56685-z

**Published:** 2024-03-15

**Authors:** Jihoon Tak, Quanxi An, Sang Gil Lee, Chang Hoon Lee, Sang Geon Kim

**Affiliations:** 1 College of Pharmacy and Integrated Research Institute for Drug Development, Dongguk University-Seoul, Goyang-si, Gyeonggi-do 10326 Republic of Korea; 2 Research and Development Institute, A Pharma Inc, Goyang-si, Gyeonggi-do 10326 Republic of Korea

**Keywords:** Gα12/Gα13, ER stress, Pyroptosis, Colitis, Inflammatory bowel disease, Biochemistry, Molecular biology, Biomarkers, Diseases

## Abstract

Inflammatory bowel disease (IBD) pathogenesis involves complex inflammatory events and cell death. Although IBD involves mainly necrosis in the digestive tract, pyroptosis has also been recognized. Nonetheless, the underlying basis is elusive. Gα12/13 overexpression may affect endoplasmic reticulum (ER) stress. This study examined how Gα12/13 and ER stress affect pyroptosis using dextran sulfate sodium (DSS)-induced colitis models. Gα12/13 levels were increased in the distal and proximal colons of mice exposed to a single cycle of DSS, as accompanied by increases of IRE1α, ATF6, and p-PERK. Moreover, *Il-6*, *Il-1β*, *Ym1*, and *Arg1* mRNA levels were increased with caspase-1 and IL-1β activation, supportive of pyroptosis. In the distal colon, RIPK1/3 levels were enhanced to a greater degree, confirming necroptosis. By contrast, the mice subjected to three cycles of DSS treatments showed decreases of Gα12/13, as accompanied by IRE1α and ATF6 suppression, but increases of RIPK1/3 and c-Cas3. AZ2 treatment, which inhibited Gα12, has an anti-pyroptotic effect against a single cycle of colitis. These results show that a single cycle of DSS-induced colitis may cause ER stress-induced pyroptosis as mediated by Gα12 overexpression in addition to necroptosis, but three cycles model induces only necroptosis, and that AZ2 may have an anti-pyroptotic effect.

## Introduction

Inflammatory bowel disease (IBD) is a persistent inflammatory condition encompassing Crohn's disease and ulcerative colitis (UC), characterized by the accumulation of inflammatory cells and disruption of the epithelial layer. The recurrence rate is high, and there is also a risk of intestinal disorders and cancer, which may affect patients’ quality of life and impose an economic burden^1^. Currently, the prevalence of IBD is on the rise annually, affecting a wide spectrum of patients with a notable lack of effective treatment^2^. Consequently, identifying new targets is imperative to overcome these limitations, which may be of help in developing effective medication. While chronic IBD predominantly entails digestive tract necrosis, there is also evidence of pyroptosis. Nevertheless, the precise cell death mechanisms remain incomplete.

Gα12 family members converge signals from G protein-coupled receptors and their expression differs depending on physiological conditions^3^; they are key molecules involved in immune responses, cellular proliferation, the regulation of inflammation, and the preservation of intestinal barrier integrity^4^. Recently, we have shown that Gα12 regulates hepatocyte ferroptosis via both ROCK1-mediated dysregulation of ALOX12 and NEMO inhibition, and that its overexpression facilitates liver injury^5,6^. In our other studies, the phosphorylation of JNK in macrophages and the induction of apoptosis by palmitate were attenuated in Gα12-deficient conditions^7,8^.

Endoplasmic reticulum (ER) stress arises from the accumulation of proteins that are unfolded or misfolded within the ER. If the ER stress exceeds the ER's protein folding capacity, it may trigger a series of events that lead to irreversible harm or cell death^9,10^. Also, research has shown the functional significance of ER stress and the unfolded protein response in maintaining intestinal homeostasis and its significant involvement in IBD pathogenesis^11^. Therefore, developing effective strategies to regulate ER stress may assist in controlling the progression of IBD. Despite the contribution of ER stress in the development of dextran sulfate sodium (DSS)-induced colitis, the molecular mechanisms of ER stress regulation in IBD, particularly during the progression of the early colitis model, have not been fully elucidated.

Programmed cell death (PCD) plays a function in defending against external infections and sustaining internal homeostasis^12^. PCD involves apoptosis, necrosis, ferroptosis, autophagy, and pyroptosis^13,14^. Of note, pyroptosis, referred to as inflammatory cell necrosis, represents a novel form characterized by its dependence on inflammatory caspases and the subsequent release of pro-inflammatory cytokines, such as interleukin-1β (IL-1β) and interleukin-18^15–17^. Also, pyroptosis factors including IL-1β, caspase-1, NOD-like receptor pyrin domain-containing protein 3 (NLRP3), and gasdermin D (GSDMD) activation may be linked to IBD pathogenesis^18^. In particular, IL-1β levels were significantly higher in the sera of UC patients than in healthy controls^19,20^. So, FL-BsAb1/17 antibody targeting IL-1β and IL-17A inhibited the development of DSS-induced UC and may have potential as a candidate for colitis treatment^21^. However, the regulators responsible for pyroptosis related to IBD have not been characterized.

The cell-death pathways and inflammation triggered by ER stress may coincide with overexpression of Gα12/13. In the present study, we initially found GPCR-related pathways upregulated in patients with UC using a public dataset, and experimentally observed overexpression of Gα12/13 in a DSS-induced mouse colitis model. Given the roles of Gα12 known in the aspects of inflammation and hepatocyte injury and its association with ER stress, we sought to explore whether Gα12/13 levels are increased in chemical-induced colitis and if so, whether their overexpression affects PCD, particularly pyroptosis, utilizing both a single cycle and three cycles of DSS-induced mouse colitis models. Moreover, as a potential anti-pyroptotic agent, we examined the ability of AZ2 to inhibit DSS-induced ER stress and pyroptosis in colons.

## Materials and methods

### Materials

Antibodies directed against Gα12 (sc-409, sc-515445), Gα13 (sc-293424), ALOX12 (sc-365194), IRE1α (sc-390960), GPX4 (sc-166570), caspase-1 (sc-56036), and F4/80 (sc-52664) were purchased from Santa Cruz Biotechnology (Santa Cruz, CA). Anti-alpha smooth muscle actin (ab7817) antibody was supplied from Abcam (Cambridge, UK), whereas anti-phospho PERK (Thr980, 3179), anti-PERK (3192), anti-ATF6 (65880), anti-RIP (3493), anti-RIP3 (D4G2A, 95702), cleaved caspase-3 (Asp175, #9664), and IL-1β (3A6, #12242) antibodies were from Cell Signaling Technology (Danvers, MA). Anti-phospho-PERK (Thr980, BS-3330R) was obtained from Bioss (Massachusetts, USA). Horseradish peroxidase-conjugated goat anti-rabbit (G-21234) and goat anti-mouse (G-21040) IgGs were provided from Invitrogen (Carlsbad, CA). DSS (160110) was obtained from MP Biomedicals (formerly ICN Biomedicals), whereas the AZ2 compound was supplied from A Pharma Co. (Goyang, South Korea). Anti-β-actin antibody (A5441) was purchased from Sigma-Aldrich (St. Louis, MO).

### Bioinformatic analysis

Gene expression data from patients, publicly available, were acquired by downloading from the Gene Expression Omnibus (GEO, https://www.ncbi.nlm.nih.gov/geo/; GSE59071). Differentially expressed genes (DEGs) were then identified using an independent *t*-test: DEGs were selected as the genes with *P*-values < 0.01 or 0.05 with absolute fold-change of > 3 or fold-change of > 1.5 or 5. The criterion for statistical significance was established at False Discovery Rate (FDR) < 0.25. The statistically enriched signaling pathways associated with clustered DEGs were systematically ranked and classified based on the ‘Gene ontology pathway’, ‘Biological process’, and ‘Reactome pathway’ using DAVID 6.8 software, DAVID Knowledgebase v2022q2, and DAVID Knowledgebase v2022q4 (https://david.ncifcrf.gov/). Each gene, symbolized as a distinct point in a volcano plot depicting significantly expressed genes, was acquired through GraphPad Prism 9.5.0.

In the context of pathway enrichment analyses, the top 10 GO terms determined by FDR-corrected *P*-value were obtained by bubble plot analysis. The gene-concept network elucidated the interplay among DEGs, and the significantly enriched ‘Biological process’ was constructed using clusterProfiler.

### DSS-induced murine colitis models

All experimental protocols were reviewed and approved by the Institutional Animal Care and Use Committee (IACUC) at Dongguk University (No. IACUC-2021-035-2). All experimental methods were performed in accordance with the ARRIVE guidelines. The C57BL/6 mice were housed in a 12 h light/dark cycle and relative humidity of 50% ± 5% under filtered, pathogen-free air, with food and water available ad libitum. Male mice at 8 weeks of age, unless otherwise indicated, were used. To minimize environmental differences, mice were maintained for at least a week before each experiment^5^.

For a single cycle of the DSS-induced colitis model, DSS was dissolved in drinking water to obtain 3% solution, and male mice were provided with 3% DSS solution to drink for 5 days. Subsequently, the mice were given normal drinking water for 5 days before being euthanized following an overnight fasting. Mice in the control group were given drinking water alone. For three cycles of the DSS-induced colitis model, mice were treated with 2.5% DSS solution to drink for 4 days, and then were given normal drinking water for another 4 days, which was repeated one more cycle (total 2 cycles). Finally, the mice were treated with 3% DSS for 5 days, followed by 5 days on normal drinking water, and were sacrificed after overnight fasting (n = 6 each). During the experiments, body weights were monitored daily, and the colon and blood samples were collected, measured, and assessed for signs of damage. Another animal group was treated with AZ2 (30 mg/kg BW, i.p.) dissolved in 0.5% carboxymethyl cellulose beginning immediately prior to the initiation of DSS treatment, and continued until the time of euthanasia (n = 6). Body weights were monitored daily.

### Cell culture and treatment

The RAW 264.7 murine macrophage and HT-29 human cell lines were purchased from the Korean Cell Line Bank (Seoul, South Korea). RAW 264.7 cells were cultured in Dulbecco’s modified Eagle’s medium (DMEM) containing 10% fetal bovine serum (FBS) and 1% penicillin–streptomycin (P/S). HT-29 cells were maintained in the RPMI-1640 containing 10% FBS and 1% P/S. To determine the effect of AZ2 on M1 macrophage, RAW 264.7 cells were seeded in 6-well plates and were incubated with LPS (100 ng/ml) for 6 h after AZ2 (10 µM) treatment for 1 h. To confirm the effect of AZ2 on cell death, HT-29 cells were seeded into 6-well culture plates and were treated with 2% DSS for 3 or 6 h after AZ2 (10 µM) treatment for 1 h.

### RNA isolation and quantitative RT-PCR assays

Total RNA was isolated employing Trizol reagent (Invitrogen, Carlsbad, CA) and subjected to reverse transcription. The resulting cDNA was subjected to amplification through qRT-PCR utilizing the LightCycler DNA Master SYBR Green-I Kit (Roche, Mannheim, Germany), as instructed by the manufacturer’s guidelines. *Gapdh* and *β-actin* served as normalization control. The primer sequences used for qRT-PCR assays are listed in Supplementary Table 1.

### Immunoblot analysis

SDS-polyacrylamide gel electrophoresis and immunoblot analyses were conducted according to the previously published procedures^22^. In brief, colon tissues were centrifuged at 3000*g* for 3 min and allowed to swell after the addition of lysis buffer in ice for 1 h. The lysates underwent centrifugation at 10,000*g* for 10 min to isolate the supernatants. Proteins were separated by 6%, 7.5%, or 12% sodium dodecyl sulfate–polyacrylamide gel electrophoresis and were transferred onto nitrocellulose membranes (Millipore, Bedford, MA). The membrane was blocked with 5% non-fat dried milk in Tris-buffered saline and Tween 20 (TBST) (20 mM Tris–HCl, 150 mM NaCl, and 0.1% Tween 20, pH 7.5) for 1 h, and incubated overnight with primary antibodies at 4 °C. After washing with TBST buffer, membranes were incubated with a horseradish peroxidase-conjugated anti-mouse IgG secondary antibody for 1 h at room temperature. The bands were detected utilizing the ECL chemiluminescence system (GE Healthcare, Buckinghamshire, UK). Equal loading of proteins was confirmed by immunoblotting for β-actin. Quantitative analyses were conducted by scanning densitometry of the immunoblots and β-actin normalization.

### Immunohistochemistry

Mouse colons were fixed in 10% formalin, embedded in paraffin, cut into sections, and mounted on slides. Tissue sections were immunostained with antibodies directed against Gα12, Gα13, or F4/80. Briefly, the paraffin-embedded sections were deparaffinized with xylene and rehydrated with alcohol series. After antigen retrieval, the endogenous peroxidase activity was quenched. The sections were pretreated with 10% normal donkey serum for 40 min to block nonspecific antibody binding and were incubated with antibodies of interest overnight at 4 °C. The sections were then treated with 2% normal donkey serum for 15 min and incubated with biotin-SP-conjugated affinity pure donkey anti-rabbit IgG for 2 h. The labeling was done by using 3,3′-diaminobenzidine. After mounting with permount solution, the sections were examined using a light microscope (DMRE, Leica Microsystems, Wetzlar, Germany), and images were acquired with Fluoview-II (Soft Imaging System GmbH, Muenster, Germany) attached to the microscope as reported previously^5,23^.

### Histopathology analysis

To assess tissue morphology, colon tissue sections were embedded in paraffin and were subjected to hematoxylin and eosin (H&E) staining using a commercially available staining kit (ScyTek Laboratories, Logan, UT, USA).

### Statistical analyses

Statistical significance was examined via two-tailed Student’s *t*-tests or one-way ANOVA coupled with Bonferroni’s method or the least significant difference (LSD) multiple comparison procedure, where appropriate. Differences were considered significant at *P* < 0.05. All experimental data were statistically analyzed using IBM SPSS Statistics 26 software or Prism version 8.0 (GraphPad Software)^5^.

## Results

### Increase of Gα12 and Gα13 levels in a single cycle of DSS-induced colitis model

**Figure 1:**
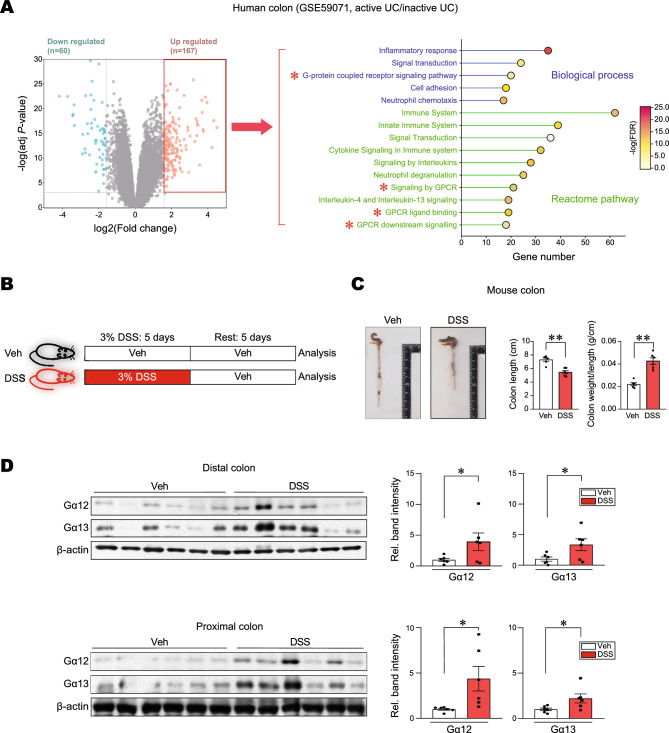
Overexpression of Gα12 and Gα13 in the colons of mice treated with a single cycle of DSS. (**A**) Analysis of RNA-seq dataset (GSE59071) from UC patients with active disease and control individuals. Volcano plots of RNA-seq data (left) (mint color, downregulated; red color, upregulated; DEGs with absolute FC > 3; and FDR is presented as a bar graph). An enrichment bubble plot of the Biological processes and Reactome pathways indicates that GPCR-related pathways were significantly upregulated in UC patients (n = 74, active UC; and n = 23, inactive UC) (right). (**B**) A scheme showing mice treatment with 3% DSS and the rest period. (**C**) The representative gross images (left), colon lengths, and the ratios of colon weight/length (right) of the DSS-treated mice (n = 6 each). (**D**) Immunoblottings for Gα12 and Gα13 in the distal and proximal colons of the mice. Band intensities represent values relative to each respective control (n = 6 each). For (**C**) and (**D**), values are expressed as mean ± SEM (*P < 0.05, **P < 0.01). Statistical significance was tested via two-tailed Student’s *t*-tests.

To identify key regulators involved in UC pathogenesis, pathways were analyzed using a public dataset (GSE59071, intestinal mucosal biopsies from UC patients with active disease and control individuals); Notably, 60 genes were significantly downregulated, whereas 167 genes were upregulated (Fig. 1A, left). Moreover, a significant proportion of upregulated genes were associated with the ‘GPCR-related pathways’ in UC patients with active disease compared to control individuals (red asterisk) (Fig. 1A, right).

Next, we employed a single cycle of DSS-induced mouse colitis models and analyzed body weight loss, colon length, and colon weight/length ratios. DSS-treated animal groups exhibited a statistically significant decrease in colon length compared to control, corroborating the presence of chemical-induced colitis (Fig. 1B,C). Given that the Gα12 family member levels vary in different cell types and organs for the regulation of physiological functions^24^ and are associated with ER stress response, we then examined Gα12 and Gα13 protein levels in the colons. As expected, Gα12/13 levels were both upregulated in the distal colon after a single cycle of DSS treatments, where colitis is usually most prominent (Fig. 1D, upper). Similar changes were also found in the proximal part (Fig. 1D, lower). These results showed overexpression of Gα12 and Gα13 in the colons of mice exposed to a single cycle of DSS treatments.

### Reversal of DSS-induced Gα12 overexpression in the distal colon by Gα12 inhibitor treatments

**Figure 2:**
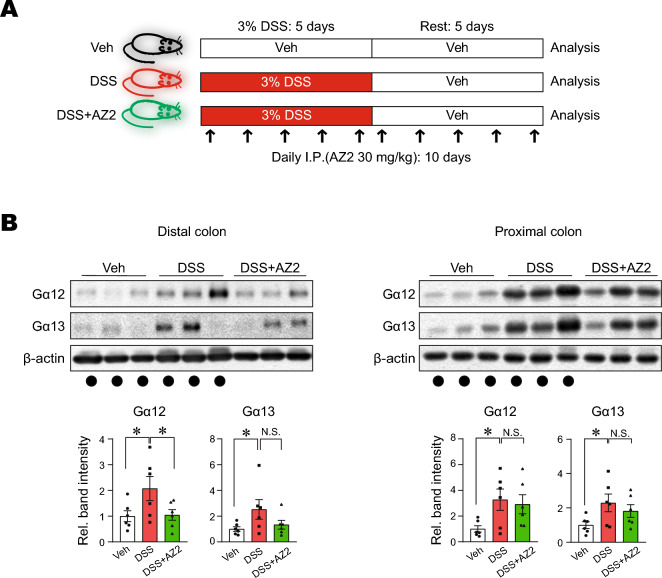
Inhibition of DSS-induced Gα12 overexpression in the distal colon by AZ2 treatments. (**A**) A scheme showing mice treatments with a single cycle of 3% DSS alone or with AZ2. Mice were treated with 3% DSS in tap drinking water for 5 days, followed by fresh tap drinking water for 5 days. In another group, mice were treated with AZ2 (30 mg/kg BW, daily i.p., 10 days) beginning with the initiation of DSS treatment and continued until the time of euthanasia. (**B**) Immunoblottings (upper) for Gα12 and Gα13 in the distal and proximal colons of the mice. Band intensities represent values relative to each respective control (n = 6 each) (lower). Experiments were done at the same time and the marked control groups (●) were shared for statistical analysis. For (**B**), values are expressed as mean ± SEM (*P < 0.05, **P < 0.01). Statistical significance was tested via one-way ANOVA coupled with the LSD multiple comparison procedure when appropriate.

A preliminary study enabled us to find AZ2 as a functional inhibitor of Gα12 and Gα13. So, we wondered whether AZ2 may have an inhibitory effect on DSS-induced overexpression of Gα12 and Gα13 in the colons (Fig. 2A). We first examined morphological changes, and found that DSS treatments alone caused statistically significant inhibition in colon lengths compared to control but that AZ2 treatments failed to affect the changes in body weight gains and colon lengths (Supplementary Fig. S1A and B). More importantly, however, AZ2 treatments almost completely inhibited Gα12 overexpression in the distal colon elicited by DSS treatments (Fig. 2B, left). However, this effect was not distinct in the proximal colon (Fig. 2B, right). AZ2 seemed to have some morphologically beneficial effects on the intestines against DSS-induced colitis. In immunohistochemistry, DSS treatment increased Gα12 expression in epithelial cells, which was reversed by AZ2 treatment. However, DSS minimally increased Gα13 levels, which was not altered by AZ2. Since inflammatory responses involve the recruitment of immune cells, colonic tissues were subjected to immunostaining for F4/80. As expected, F4/80-positive cells were increased, but this effect was not affected by AZ2. The data support the notion that Gα12 may play a role in epithelial cells (Supplementary Fig. S2). Our results show that AZ2 inhibited Gα12 and Gα13 overexpression, particularly in the distal colons of mice exposed to a single cycle of DSS treatments, which may be associated with a pharmacological effect in colons against the DSS-induced colitis.

### Inhibition of IRE1α induction by AZ2 treatments in a single cycle of colitis model

**Figure 3:**
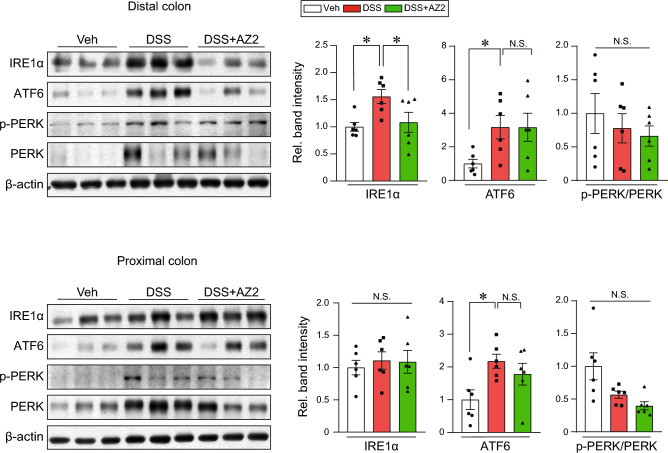
Suppression of IRE1α induction in the colons of mice treated with a single cycle of DSS with AZ2 treatments. Immunoblottings (left) for representative ER stress markers in the distal and proximal colons of the same samples as in Fig. 2A. Band intensities represent values relative to each respective control (n = 6 each) (right). Values are expressed as mean ± SEM (*P < 0.05, **P < 0.01). Statistical significance was tested via one-way ANOVA coupled with the LSD multiple comparison procedure when appropriate.

Given the finding of Gα12 inhibition by AZ2 treatments in a single cycle model and the association of Gα12 overexpression with ER stress^5^, we next examined ER stress marker levels in the same samples. As expected, canonical ER stress markers including IRE1α and ATF6 were markedly enhanced in both distal and proximal colons after a single cycle of DSS treatments, whereas p-PERK levels were weakly changed (Fig. 3). In addition, AZ2 treatments caused a decrease in IRE1α band intensities in the distal colon although ATF6 and p-PERK levels were not significantly affected (Fig. 3, upper). AZ2 treatments had no or minimal effects on the ER stress markers in the proximal colon (Fig. 3, lower). These results show that a single cycle of DSS treatments caused ER stress in both distal and proximal colons and that AZ2 might have effectiveness in inhibiting IRE1α overexpression in the distal colons of mice exposed to a single cycle of DSS treatments.

### Lack of inhibition of necroptosis markers by AZ2 treatments in a single cycle of colitis model

**Figure 4:**
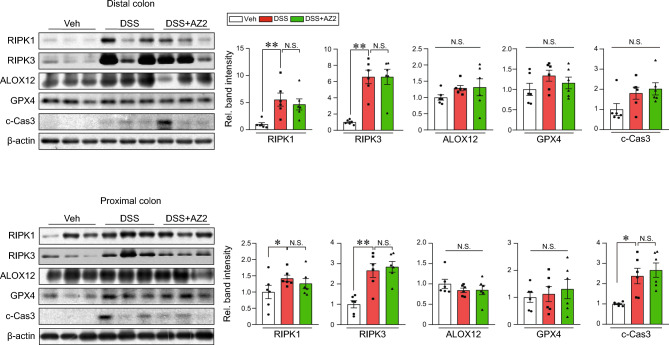
Necroptosis and ferroptosis marker levels in the colons of mice treated with a single cycle of DSS alone or with AZ2 treatments. Immunoblottings (left) for representative necroptosis and ferroptosis markers in the distal and proximal colons of the same samples as in Fig. 2A. Band intensities represent values relative to each respective control (n = 6 each) (right). Values are expressed as mean ± SEM (*P < 0.05, **P < 0.01). Statistical significance was tested via one-way ANOVA coupled with the LSD multiple comparison procedure when appropriate.

In a subsequent experiment, we wondered whether overexpression of Gα12 and Gα13 in conjunction with increases in ER stress markers is associated with cell death pathways. First, we examined representative necroptosis and ferroptosis marker levels and found that RIPK1 and RIPK3 levels were substantially increased in both distal and proximal colons, supportive of necroptosis, whereas those of ferroptosis markers including ALOX12 and GPX4 were only minimally increased (i.e., statistically insignificant) (Fig. 4). c-Cas3 was also significantly enhanced in the proximal colon. Treatment with AZ2 was ineffective in inhibiting necroptosis and ferroptosis marker levels. Our results showed the inducible effect of a single cycle of DSS treatments on necroptosis and the lack of AZ2 efficacy on the cell death pathway.

### Inhibition of pyroptosis by AZ2 treatments in a single cycle of colitis model

**Figure 5:**
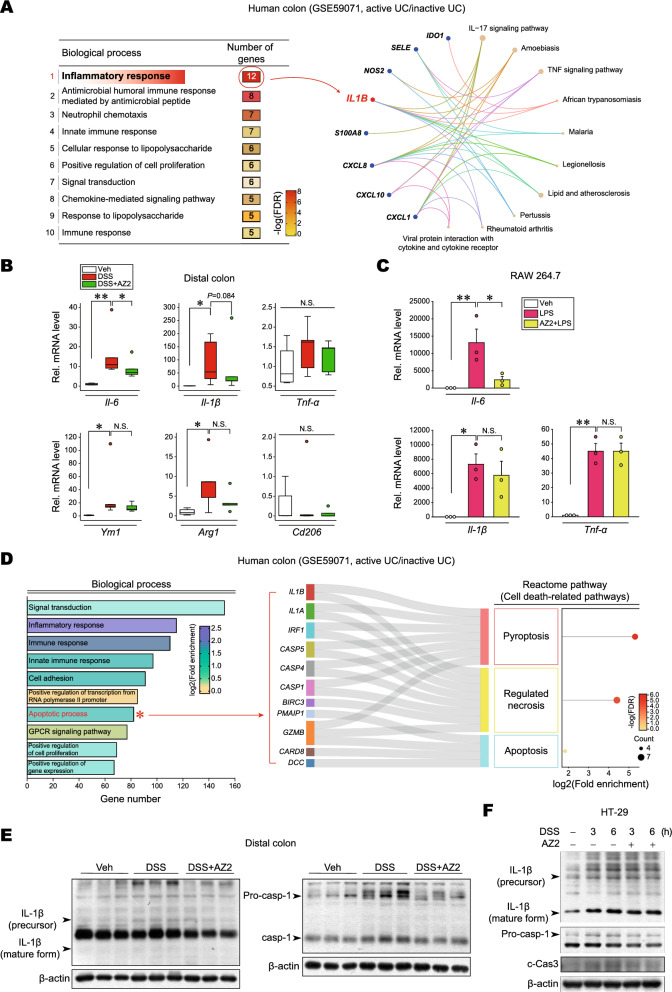
Inhibition of DSS-induced pyroptosis in the distal colon by AZ2 treatments. (**A**) Biological process analysis using RNA-seq dataset (GSE59071) obtained from UC patients with active disease and control individuals. The inflammatory response pathway is indicated in red (left). Square boxes display FDR values (n = 74; active UC, n = 23; inactive UC, FC > 5). The gene-concept network (cnetplot) of functional GO enrichment analyses (right) from the leading genes was highlighted in the circles’ red areas in the first biological process rank. The cnetplot depicts gene and pathway analysis linkages as a network. Circle size indicates genes represented in a given pathway analysis. (**B**) Real-time RT-PCR assays for *Il-*6, *Il-1β*, *Tnf-α*, *Ym1*, *Arg1*, and *Cd206* in the distal colons of the same samples as in Fig. 2A. The horizontal lines within the boxes show the median value, 5%-95% percentile (ends of the boxes), and range of minimum to maximum values (whiskers). Each dot indicates outlier values. (**C**) Real-time RT-PCR assays for *Il-*6, *Il-1β*, and *Tnf-α* in RAW 264.7 cells treated with LPS (100 ng/ml, 6 h) after AZ2 treatment (10 µM, 1 h) (n = 3 each). (**D**) Biological process analysis using RNA-seq dataset (GSE59071) from UC patients with active disease and control individuals. The apoptotic process pathway is indicated in red (left). Pyroptosis, necrosis, and apoptosis (i.e., cell death-related) pathways were based on the Sankey diagram (plot) of DEGs using colon transcriptome data from UC patients with active disease and control individuals (right). The Sankey diagram represents genes within each pathway; dot plots with sizes indicate gene numbers and dot colors display FDR values (n = 74; active UC, n = 23; inactive UC, FC > 1.5). (**E**) Immunoblottings for representative pyroptosis markers in the distal colons of the same samples as in Fig. 2A. (**F**) Immunoblottings for pyroptosis and apoptosis markers. HT-29 cells were treated with 2% DSS for 3 or 6 h after AZ2 treatment (10 µM, 1 h) (n = 3; repeated three times with similar results). For (**B**) and (**C**), values are expressed as mean ± SEM (*P < 0.05, **P < 0.01). Statistical significance was tested via one-way ANOVA coupled with the LSD multiple comparison procedure when appropriate.

As a continuing effort to find the functional role of Gα12/13 overexpression and IRE1α induction in the colon by a single cycle of DSS treatments, we next determined whether Gα12 and ER stress induction may affect pyroptosis. First, we tried to identify the ‘inflammatory response’ biological processes using DAVID database analysis for UC patients with active disease and found that *IL-1β* is a core gene in the gene-concept network (Fig. 5A). In the assays of RT-PCR for inflammatory mediators, *Il-6*, *Il-1β*, *Ym1*, and *Arg1* transcript levels were increased in the distal colon by a single cycle of DSS treatments. In addition, AZ2, an agent that inhibits Gα12 and IRE1α overexpression against DSS, was effective in inhibiting *Il-6* and, to a lesser degree *Il-1β*, supportive of its anti-pyroptotic effect against a single cycle of DSS treatments (Fig. 5B)*.* Likewise, treatment of RAW 264.7 cells with AZ2 prevented LPS from increasing the transcript levels of *Il-6,* but not *Il-1β*, and *Tnf-α* (Fig. 5C), being consistent with the result in animal experiments. When we performed the GO analysis using the same public dataset, we found that the apoptotic process pathway has significantly higher gene numbers in UC patients with active disease compared to control individuals (Fig. 5D, left). The apoptotic process pathway consisted of 82 genes associated with ‘Pyroptosis’, ‘Regulated necrosis’, and ‘Apoptosis’ in the Sankey diagram visualization (Fig. 5D, right), suggestive of *IL-1β*-mediated cell death in UC patients.

To confirm pyroptosis in a single cycle of DSS-induced colitis, we further examined the protein markers for pyroptosis (i.e., IL-1β and caspase-1), and found the expected changes in IL-1β and caspase-1 levels (Fig. 5E). The precursor forms for IL-1β and caspase-1 were also increased, indicative of adaptive increases after multiple days of DSS exposures and compensatory changes after resting period. Of note, AZ2 was active in attenuating these changes, fortifying its pharmacological ability to inhibit Gα12 and IRE1α in conjunction with an anti-pyroptic effect. Similarly, DSS treatment increased IL-1β, caspase-1, and c-Cas3 levels in HT-29 cells, which was inhibited by AZ2 (Fig. 5F). Together, our results provide evidence that a single cycle of DSS-induced colitis may result in pyroptosis in the distal colon and that AZ2 may be pharmacologically active in this event.

### Lack of Gα12/13 overexpression by 3 cycles of DSS treatments, a model inducing only necroptosis

**Figure 6:**
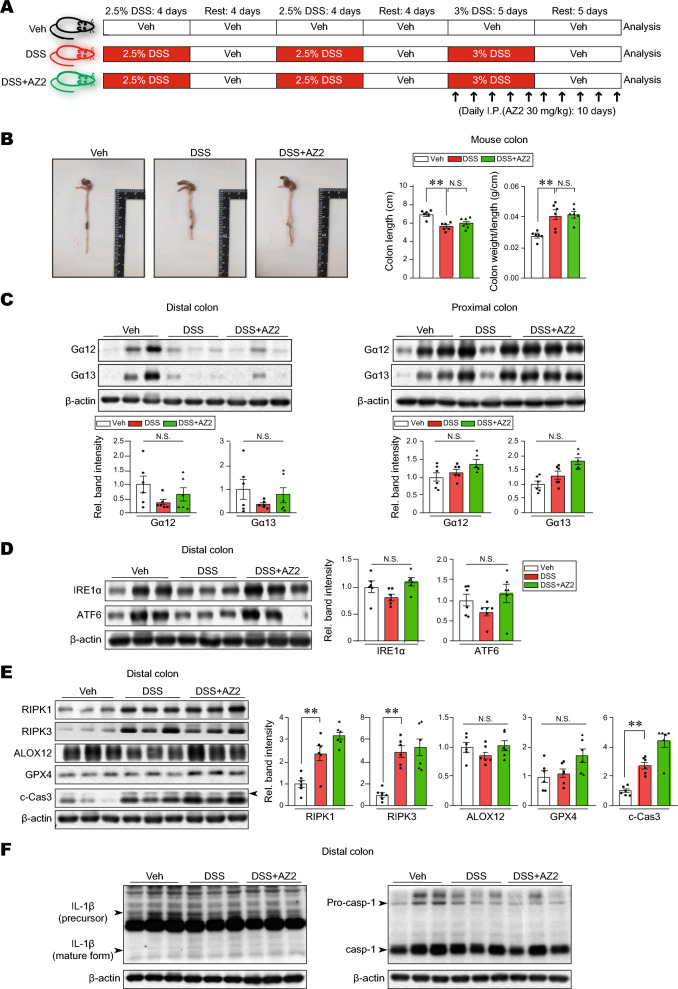
Gα12/Gα13, ER stress, necroptosis, ferroptosis, and pyroptosis marker levels in mice treated with three cycles of DSS alone or with AZ2 treatments. (**A**) A scheme showing mice treatments with three cycles of 3% DSS alone or with AZ2. Mice were treated with 2.5% DSS in tap drinking water for 4 days, followed by fresh tap drinking water for 4 days. After the second cycle, the mice were treated with 3% DSS for 5 days and switched to fresh drinking water for 5 days. In another group, mice were treated with AZ2 (30 mg/kg BW, daily i.p., 10 days) beginning with the onset of the third cycle. (**B**) The representative gross images (left), colon lengths, and the ratios of colon weight/length (right) in mice treated with three cycles of DSS alone or with AZ2 treatments (n = 6 each). (**C**) Immunoblottings (upper) for Gα12 and Gα13 in the distal and proximal colons of the same samples as in (**B**). Band intensities represent values relative to each respective control (n = 6 each) (lower). (**D**) Immunoblottings (left) for IRE1α and ATF6 in the distal colons of the same samples as in (**B**). Band intensities represent values relative to each respective control (n = 6 each) (right). (**E**) Immunoblottings (left) for representative necroptosis and ferroptosis markers in the distal colons of the same samples as in (**B**). Band intensities represent values relative to each respective control (n = 6 each) (right). (**F**) Immunoblottings for representative pyroptosis markers in the distal colons of the same samples as in (**B**). For (**B**–**E**), values are expressed as mean ± SEM (*P < 0.05, **P < 0.01). Statistical significance was tested via one-way ANOVA coupled with the LSD multiple comparison procedure when appropriate.

Having identified the occurrence of pyroptosis in a single cycle of DSS-induced colitis, we wondered whether more severe multiple cycles of DSS treatments had a similar effect. As expected, 3 cycles of the DSS-induced IBD model showed a significant decrease in colon length and body weight gain with pathological alterations (Fig. 6A,B, and Supplementary Fig. S3A). AZ2 treatments failed to exhibit any beneficial effects on the morphological changes (Fig. 6B and Supplementary Fig. S3A). Of note, 3 cycles of DSS treatments caused a lack of Gα12/13 overexpression; rather, Gα12 and Gα13 levels seemed to be diminished, especially Gα12 in the distal colon (Fig. 6C). Consistently, the levels of ER stress markers including IRE1α and ATF6 tended to decrease in the distal colon (Fig. 6D). However, the levels of necroptosis markers such as RIPK1, RIPK3 and c-Cas3 levels were all significantly increased in the distal colons, confirmative of necroptosis. Ferroptosis markers including ALOX12 and GPX4 not much changed (Fig. 6E). By the same token, AZ2 treatments had no beneficial effect. We also assessed the levels of pyroptosis markers (IL-1β and caspase-1) and found no changes in the distal colon (Fig. 6F). Finally, repeated DSS treatments increased fibrotic marker (α-SMA) levels, which were not changed by AZ2 treatments (Supplementary Fig. S3B). Overall, our results demonstrate that multiple cycles of DSS treatments resulted in necroptosis, but not pyroptosis, especially in the distal colon, and that this event had no relationship with Gα12/Gα13 and ER stress marker level changes.

## Discussion

The intricate pathogenesis of chronic inflammatory gastrointestinal disorder arises from a combination of genetic and environmental factors, and the dynamic interaction between the host's immune response and intestinal microbiota^25–27^. Gα12 and Gα13 have been established as mediators of signals from GPCRs to Rho GTPase activation^28^. Previously, we showed that Gα12 was overexpressed in hepatocytes and hepatic stellate cells in the context of acute inflammatory responses and fibrogenesis^5,29^. Also, Gα13 ablation not only controls myosin expression but also facilitates oxidative metabolism, which could correspond to the processes associated with fiber type conversion^30^. Recently, we reported that Gα12 overexpression by ER stress facilitates hepatocyte ferroptosis through ROCK1-mediated dysregulation of ALOX12, and miR-15a^5^.

Analysis of primary intestinal epithelial cells (IEC) from IBD patients revealed an augmented expression of Rho-GDP dissociation inhibitor α^31^. Furthermore, early apoptotic cells activate Rho in adjacent viable cells, leading to the extrusion of apoptotic cells from the epithelium^32^. Activation of the RhoA/ROCK pathway stimulates TNF-α and IL-1β production, with a positive correlation observed between RhoA and TNF-α in the inflammatory tissues of Crohn's disease patients^33^. In an investigation, pro-inflammatory cytokines including IL-1β, IL-6, and IFN-γ exhibited increased levels with prominent polyps found in the distal colon, while TNF-α showed heightened expression in the proximal part^34,35^. There is yet no previous research investigating the site-specific expression patterns and the role of Gα12/13 in chemical-induced colitis models. Since the differential inflammatory responses may be associated with DSS-induced colitis^36^, this study explored changes in Gα12/13 levels and their association with distinct inflammatory processes. An important finding of our study is that Gα12/13 levels were increased in the distal and proximal colons of mice after a single cycle of DSS treatments. Here, our findings confirmed these changes along with colon length shortening.

A series of studies have shown that ER stress contributes to the pathogenesis of UC, and effective reduction of excessive ER stress may offer enhanced therapeutic potential for colitis treatment^37,38^. In the conditions of ER stress, the extent of activation within the UPR pathways determines whether cells restore homeostasis, trigger inflammation, or initiate cell death programs. Importantly, it's worth noting that the three distinct UPR pathways may have varying roles in specific colon regions, and may not necessarily be activated concurrently during the development of IBD^39^. In the present study, a single cycle of DSS treatments caused high expression of IRE1α in the distal colon, whereas ATF6 was overexpressed in both distal and proximal parts. Another finding from our laboratory showed that IRE1α activation leads to Gα12 overexpression which may add to catastrophic cell death like ferroptosis^5^. Nonetheless, other forms of necrosis may also be controlled by the pathway.

Having previously reported DSS-induced ER stress and inflammatory response in intestines^40^, we wondered whether the AZ2 compound inhibits ER stress in DSS-induced colitis. Interestingly, AZ2 treatments repressed IRE1α activation, but not that of ATF6 and p-PERK, raising the possibility that AZ2 has a specific effect on IRE1α. This contention is in line with our report that IRE1α regulates Gα12 under ER stress conditions^5^, fortifying the ability of AZ2 to functionally inhibit Gα12. Additionally, the specific inhibitory effect of AZ2 on Gα12 may account for its anti-inflammatory efficacy in the distal colon. In this respect, the divergent gene expression profiles in the distal and proximal colon influenced by the AZ2 during inflammation could hold significant implications. The lack of significant Gα13 recovery by AZ2 treatments may be due to isoform-specific regulatory mechanisms within the colon.

An intriguing finding of our study is the discovery of IL-1β and caspase-1 activation by DSS-induced colitis, which accounts for pyroptosis. Of the inflammatory mediators, we found IL-1β as a central molecule in a single cycle of the DSS-induced colitis model. Along with increases in IL-1β mRNA, we confirmed increases in the pro-IL-1β and IL-1β levels in parallel with Gα12 overexpression. In addition, our findings supported an increase in pro-caspase-1 and cleaved caspase-1 levels although the change in the cleaved form was not very distinct. Hence, colonic inflammation observed in a single-cycle model may promote pyroptosis in colons, particularly more in distal colons in association with Gα12 overexpression. Based on the knowledge that activation of inflammasomes and maturation of pro-IL-1β to IL-1β by active caspase-1 induce pyroptosis^37^, we raised the notion that Gα12 axis may regulate pro-caspase-1/pro-IL-1β overexpression and their processing which facilitates pyroptosis. Moreover, treatments with AZ2 resulted in the inhibition of IL-1β and caspase-1, suppressing pyroptosis. Additional studies remain to examine the identified targets in the milder colitis model because 3% DSS may be prone to cause severe cell death.

Pro-IL-1β and pro-IL-18 are cleaved to generate mature IL-1β and IL-18. Thus, blocking IL-1β and IL-18 would be one of the plausible approaches for the treatment of colitis accompanying pyroptosis. In addition, the cytotoxic N-terminal fragment of GSDMD is overexpressed in both patients with IBD and those with experimental colitis^41^. It is also involved in the formation of membrane pores, and IL-1β and IL-18 are released through these pores formed during pyroptosis^42^. Moreover, it can be inferred that aside from pyroptosis, various factors, including genetic factors, immune response, and intestinal microbiota, are implicated in cell death in the chronic DSS-induced colitis model.

Elevated levels of cell death have been reported in the epithelium of patients with both UC and CD^43,44^. Notably, necroptosis can induce inflammatory responses and alter cell membrane permeability in intestinal epithelial cells. As a key contributor to necroptosis, RIPK3 exacerbates inflammatory responses and cell membrane permeability in the patients^45^. Additionally, RIP3 can signal necroptosis independently of RIP1, promoting pro-inflammatory cytokine production. RIPK3 has also been shown to promote NLRP3 inflammasome and IL-1β inflammatory responses^46^. Consequently, in the distal part of the acute model, we observed an increase in pyroptosis markers, IL-1β, and caspase-1.

Severe or prolonged ER stress can activate cell death signaling^13,47,48^. There are alternative forms of programmed cell death, including ferroptosis, necroptosis, and pyroptosis, contributing to the development of intestinal diseases^49,50^. It has been shown that mice lacking Mlkl exhibited interrelation with enhanced expression of ferroptosis marker during ischemia–reperfusion injury^51^, implying that ferroptosis might be attenuated in situations where necroptosis occurs. In the present study, we probed into the mechanistic underpinnings of Gα12 levels elicited by 3 cycles of DSS treatments and found that Gα12 and Gα13 levels showed a decreasing tendency in the 3 cycles model. More importantly, necroptosis markers were highly upregulated in the distal part, whereas those of pyroptosis and ferroptosis were largely unaffected, suggesting that an unexpected shift may occur between pyroptosis and necroptosis.

In table 1, we present a summary of the expression patterns of Gα12, Gα13, ER stress markers, and associated cell death markers across proximal and distal segments: necroptosis markers RIPK1, RIPK3; ferroptosis markers ALOX12, GPX4; pyroptosis markers IL-1β, caspase-1; and the apoptotic cell death marker (cleaved caspase-3). In the distal part of a single-cycle colitis model, upregulation of ER stress markers was observed alongside increases of Gα12 and Gα13, as well as markers associated with necroptosis and pyroptosis. However, more severe conditions like three cycles of DSS-induced colitis seem to result in necroptosis irrespective of Gα12 and Gα13. In the distal part of the multiple cycle model, Gα12 exhibited a rather decrease, while other markers showed no changes, except for the necroptosis marker RIPK1 and RIPK3. Our results support the lack of beneficial effects of AZ2 in this model.

**Table 1:** A summary of the outcomes in 1 and 3 cycle(s) of DSS-induced colitis models.

Targets	1 Cycle IBD model		3 Cycles IBD model	
	Distal	Proximal	Distal	Proximal
Gα12	Increase	Increase	Decreasing trend	No change
Gα13	Increase	Increase	Decreasing trend	No change
ER stress markers	Distal	Proximal	Distal	Proximal
IRE1α	Increase	No change	No change	No change
ATF6	Increase	Increase	No change	No change
p-PERK/PERK	No change	No change	No change	No change
Cell death markers	Distal	Proximal	Distal	Proximal
RIPK1	Increase	Increase	Increase	No change
RIPK3	Increase	Increase	Increase	Increase
ALOX12	No change	No change	No change	Increasing trend
GPX4	No change	No change	No change	No change
c-Cas3	No change	Increase	Increase	Increase
IL-1β	Increase	–	No change	–
Caspase-1	Increase	–	No change	–

In our GEO data analysis of UC patients, “Pyroptosis”, “Regulated necrosis”, and “Apoptosis” were positively correlated with UC patients, which supports our result showing increased expression of RIPK1 and RIPK3, but not ALOX12 and GPX4, in the acute colitis model. Our findings also showed that G protein coupling to a variety of GPCRs might be overexpressed in patients with UC, which is consistent with our data. Recently, studies have identified distinct microbiota diversity and community structures in colorectal cancer (CRC), leading to significant differences in tumor diameter^52^. In the analysis of the microbiome associated with CRC, *Bacteroidetes Cluster 2* and *Pathogen Cluster* were more prevalent in distal malignancies, whereas *Prevotella Cluster* and *Firmicutes Cluster 2* were more abundant in proximal cancer patients^53^. The existing literature on microbiota, coupled with our results showing differential expression patterns of Gα12, Gα13, and death markers, may account for the distinction between acute and chronic colitis. Overall, the outcomes of this research provide evidence that a single cycle of DSS-induced colitis may cause ER stress-induced pyroptosis as mediated by Gα12 overexpression in addition to necroptosis, but three cycles model induces only necroptosis, and that AZ2 may have a protective effect against pyroptosis.

### Supplementary Information

Supplementary Information.

## Data Availability

The datasets used and/or analyzed during the current study are available from the corresponding author on reasonable request. Supplementary data are available in Supplementary Information.
