# Supplementary material for: Gα12 and endoplasmic reticulum stress-mediated pyroptosis in a single cycle of dextran sulfate-induced mouse colitis
Source: Sci Rep. 2024 Mar 15;14:6335. doi: 10.1038/s41598-024-56685-z (PMC10943197; doi:10.1038/s41598-024-56685-z)
Supplement: Supplementary file 1 — Supplementary Information. [file 41598_2024_56685_MOESM1_ESM.docx]

***Supplementary Information***

Gα12 and endoplasmic reticulum stress-mediated pyroptosis in

a single cycle of dextran sulfate-induced mouse colitis

Jihoon Tak, Quanxi An, Sang Gil Lee, Chang Hoon Lee, and Sang Geon Kim

**Table of contents**

Supplementary figure.…………………………………………………………………………….........**2**

Supplementary table……………………………………………………………………………………**5**

**Supplementary Figure S1. Body weight and colon length changes in the mice treated with a single cycle of DSS alone or with AZ2**

**(A)** Body weight (left) and the percent weight (right) changes of mice were measured in the same mice as in **Fig. 2A**.

**(B)** The representative gross images (left), length, and the ratio of weight/length of colons (right) were assessed using the same mice as in **Fig. 2A**. Experiments were done at the same time and the marked control groups (●) were shared for statistical analysis. Representative images were shown.

For **A** and **B**, values are expressed as mean ± SEM (**P* < 0.05, ***P* < 0.01). Statistical significance was tested via one-way ANOVA coupled with the LSD multiple comparison procedure when appropriate.

**Supplementary Figure S2. H&E staining and immunohistochemistry in the proximal colons of mice exposed to DSS with or without AZ2 treatments**

Histopathology and immunohistochemistry for Gα12, Gα13, and F4/80 were assessed in the proximal colons of the mice used as in **Fig. 2A** (n = 6 each). Experiments were done at the same time and the marked control groups (●) were shared for comparison. Representative images were shown.

**Supplementary Figure S3. Body weight and α-SMA level changes in mice exposed to three cycles of DSS treatments alone or with AZ2 treatments.**

**(A)** Body weight (left) and the percent weight (right) changes of mice were measured in the same mice as in **Fig. 6B**.

**(B)** Immunoblottings for α-SMA in the same samples as in **Fig. 6B**.

For **A**, values are expressed as mean ± SEM (**P* < 0.05, ***P* < 0.01). Statistical significance was tested via one-way ANOVA coupled with Bonferroni’s method.

**Supplementary Table 1. The sequences of primers**

| **Genes symbols** | **Forward** | **Reverse** |
| --- | --- | --- |
| *Il1b* | GGAGAACCAAGCAACGACAAAATA | TGGGGAACTCTGCAGACTCAAAC |
| *Tnfα* | TACTGAACTTCGGGGTGATCGGTCC | CAGCCTTGTCCCTTGAAGAGAACC |
| *Il6* | TTCCATCCAGTTGCCTTCTT | ATTTCCACGATTTCCCAGAG |
| *Arg1* | CTCCAAGCCAAAGTCCTTAGAG | AGGAGCTGTCATTAGGGACATC |
| *Cd206* | CTCTGTTCAGCTATTGGACGC | CGGAATTTCTGGGATTCAGCTTC |
| *Ym1* | CAGGTCTGGCAATTCTTCTGAA | GTCTTGCTCATGTGTGTAAGTGA |
| *Gapdh* | AACGACCCCTTCATTGAC | TCCACGACATACTCAGCAC |
| *β-actin* | CTGAGAGGGAAATCGTGCGT | TGTTGGCATAGAGGTCTTTACGG |
